# Detection of autism spectrum disorder-related pathogenic trio variants by a novel structure-based approach

**DOI:** 10.1186/s13229-024-00590-9

**Published:** 2024-04-03

**Authors:** Sadhna Rao, Anastasiia Sadybekov, David C. DeWitt, Joanna Lipka, Vsevolod Katritch, Bruce E. Herring

**Affiliations:** 1 Department of Biological Sciences, Neurobiology Section, Dornsife College of Letters, Arts and Sciences, University of Southern California, Los Angeles, CA 90089 USA; 2 Quantitative and Computational Biology, University of Southern California, Los Angeles, CA 90089 USA; 3 Department of Chemistry, University of Southern California, Los Angeles, CA 90089 USA; 4 Neuroscience Graduate Program, University of Southern California, Los Angeles, CA 90089 USA; 5 Department of Pathology, Genentech, Inc., South San Francisco, CA 94080 USA; 6 Department of Neuroscience, Genentech, Inc., South San Francisco, CA 94080 USA

**Keywords:** Glutamatergic neurotransmission, Autism spectrum disorders, Synaptic dysfunction, Missense mutations, TRIO-related disorders, Mutation modeling

## Abstract

**Background:**

Glutamatergic synapse dysfunction is believed to underlie the development of Autism Spectrum Disorder (ASD) and Intellectual Disability (ID) in many individuals. However, identification of genetic markers that contribute to synaptic dysfunction in these individuals is notoriously difficult. Based on genomic analysis, structural modeling, and functional data, we recently established the involvement of the TRIO-RAC1 pathway in ASD and ID. Furthermore, we identified a pathological *de novo* missense mutation hotspot in TRIO’s GEF1 domain. ASD/ID-related missense mutations within this domain compromise glutamatergic synapse function and likely contribute to the development of ASD/ID. The number of ASD/ID cases with mutations identified within TRIO’s GEF1 domain is increasing. However, tools for accurately predicting whether such mutations are detrimental to protein function are lacking.

**Methods:**

Here we deployed advanced protein structural modeling techniques to predict potential *de novo* pathogenic and benign mutations within TRIO’s GEF1 domain. Mutant TRIO-9 constructs were generated and expressed in CA1 pyramidal neurons of organotypic cultured hippocampal slices. AMPA receptor-mediated postsynaptic currents were examined in these neurons using dual whole-cell patch clamp electrophysiology. We also validated these findings using orthogonal co-immunoprecipitation and fluorescence lifetime imaging (FLIM-FRET) experiments to assay TRIO mutant overexpression effects on TRIO-RAC1 binding and on RAC1 activity in HEK293/T cells.

**Results:**

Missense mutations in TRIO’s GEF1 domain that were predicted to disrupt TRIO-RAC1 binding or stability were tested experimentally and found to greatly impair TRIO-9’s influence on glutamatergic synapse function. In contrast, missense mutations in TRIO’s GEF1 domain that were predicted to have minimal effect on TRIO-RAC1 binding or stability did not impair TRIO-9’s influence on glutamatergic synapse function in our experimental assays. In orthogonal assays, we find most of the mutations predicted to disrupt binding display loss of function but mutants predicted to disrupt stability do not reflect our results from neuronal electrophysiological data.

**Limitations:**

We present a method to predict missense mutations in TRIO’s GEF1 domain that may compromise TRIO function and test for effects in a limited number of assays. Possible limitations arising from the model systems employed here can be addressed in future studies. Our method does not provide evidence for whether these mutations confer ASD/ID risk or the likelihood that such mutations will result in the development of ASD/ID.

**Conclusions:**

Here we show that a combination of structure-based computational predictions and experimental validation can be employed to reliably predict whether missense mutations in the human *TRIO* gene impede TRIO protein function and compromise TRIO’s role in glutamatergic synapse regulation. With the growing accessibility of genome sequencing, the use of such tools in the accurate identification of pathological mutations will be instrumental in diagnostics of ASD/ID.

**Supplementary Information:**

The online version contains supplementary material available at 10.1186/s13229-024-00590-9.

## Background

Autism Spectrum Disorder (ASD) is a heterogeneous neurodevelopmental disorder that affects 2–3% of the western population [1]. Large-scale population-based cohort studies, the high recurrence of ASD within siblings, and the discovery of several genetic risk factors have shown that 5–20% of ASD cases have identifiable genetic etiology and are frequently comorbid with Intellectual Disability (ID) [2–5]. Scientific progress in recent years has produced rapid advances in human whole-exome sequencing and the discovery of more ASD/ID risk-genes [6–9]. In particular *de novo* variants (i.e. new variants that arise from spontaneous germline mutations) confer five-fold higher risk than commonly inherited variants, and may contribute to 15–20% of population-wide ASD-risk [5, 10]. These results, along with a demonstrated increase in diagnostic yield from genetic testing have influenced diagnostic recommendations for ASD [11, 12]. While behavioral testing is the basis for diagnostic evaluation, the case for inclusion of genetic testing for ASD/ID in healthcare practice standards and guidelines is gaining momentum [13–18]. The ability to identify pathogenic mutations in individual genes that contribute to patient symptomatology stands to uncover specific syndromes within larger populations of individuals with neurodevelopmental disorders. The identification of such syndromes will be invaluable to clinical genetic testing and the development of personalized therapeutic interventions for treating these disorders.

In the past decade, exome sequencing studies have detected a strong enrichment of ASD-related *de novo* mutations in synaptic regulatory genes suggesting that glutamatergic synapse dysfunction is one of the primary contributing factors to the development of ASD [19]. We recently discovered a hotspot of ASD-related missense mutations in *TRIO*, the gene encoding the glutamatergic synapse regulatory protein TRIO [20]. These missense mutations are clustered within the region of the *TRIO* gene that encodes the GEF1 domain of the TRIO protein. Several other reports have characterized TRIO-GEF1 domain mutations and found deleterious effects on RAC1 function in various model systems [21–23]. TRIO’s GEF1 domain binds to and activates the small GTPase, RAC1. Through its ability to activate RAC1, TRIO promotes the polymerization of actin at glutamatergic synapses and exerts an influence on glutamatergic synapse function [24]. The hotspot of *de novo* mutations in TRIO’s GEF1/DH1 domain we previously identified exhibits more ASD-associated missense mutations per sequence base than well-known ASD genes such as *SCN2A, SYNGAP and SHANK2* [20]. None of the disruptive mutations in *TRIO* within the region encoding the GEF1 domain were present in family member controls [20, 25–28], or found in the Genome Aggregation Database (gnomAD) of control genomes [29]. Our structural analysis suggested that these ASD-related missense mutations in TRIO either interfere with conformational stability of the GEF1 domain or disrupt the GEF1/RAC1 interface. ASD-associated mutations within GEF1/DH1 predicted to interfere with RAC1 activation were experimentally confirmed to impact glutamatergic neurotransmission [20]. Given the strength of this method in predicting disruptive mutations in *TRIO*, we reasoned that our structure-based approach might be able to effectively determine whether new missense variants in TRIO’s GEF1 domain are detrimental to TRIO protein function.

In this study, we employed structure-based modeling to predict mutations deleterious to TRIO function and used organotypic slice electrophysiology to test whether the mutations affect TRIO’s influence on glutamatergic neurotransmission. This combination of computational predictions and experimental validation allowed us to identify new *TRIO* variants that disrupt TRIO-RAC1 signaling, compromise synapse function, and are likely to confer high risk for ASD/ID. Specifically, our approach uses structural modeling to predict the effect of mutations within TRIO’s catalytic GEF1 domain that forms the TRIO-RAC1 interface, on the stability and binding of the TRIO-RAC1 complex. The method was validated by experimentally testing the effect of these mutations on glutamatergic transmission in rodent neurons in vitro. Experimental validation of eight mutations predicted as disruptive showed a 75% prediction success rate. Control mutations predicted to be benign were found to have no impact on TRIO function, placing overall accuracy for all mutations at 80%. As more individuals with ASD/ID are identified with mutations in TRIO’s GEF1 domain, this combination of *in silico* prediction with in vitro validation can provide a fast and reliable approach to screen these mutations, and predict a potential contribution to synaptic dysfunction in ASD/ID.

## Methods

### Structure-based computational predictions

The effects of mutations on stability and binding were predicted using MERSI protocol [30] as described in Sadybekov et al. 2017 [20]. Calculations were performed using ICM molecular modeling software (Molsoft LLC). We used the high-resolution crystal structure of TRIO-GEF1 in complex with RAC1 (PDB code: 2NZ8) to model the interactions, with the all-atom model of TRIO-GEF1 generated by the ICM conversion algorithm that adds and optimizes hydrogens and optimizes His, Asn and Gln side chain isomers. Specifically, energy optimization of mutant protein side chain conformations in 8 Å proximity of the mutation was performed using a biased probability Monte Carlo algorithm. The free energy change in protein stability ∆∆G_*stability*_ (1) and protein binding ∆∆G_*binding*_ (2) was then calculated as a difference in folding or binding free energies of mutant and WT protein:

1 $$\varDelta \varDelta {G}_{stability}=\left(\varDelta {G}_{folded}^{mutant}- {\varDelta G}_{unfolded}^{mutant}\right)-(\varDelta {G}_{folded}^{WT}- {\varDelta G}_{unfolded}^{WT})$$

2 $$\varDelta \varDelta {G}_{binding}=\varDelta {G}_{binding}^{mutant}- {\varDelta G}_{binding}^{WT}$$

As per MERSI protocol, the free energy of the unfolded states was approximated by a sum of the residue-specific energies, derived empirically using a large set of experimental data. A positive free energy ∆∆G value indicates that the mutation is likely to be destabilizing.

### Experimental constructs

Human *TRIO-9* (or TRIO-9s in McPherson CE et al., 2004) was generated from a TRIO-FL cDNA generously provided by Dr. Betty A. Eipper (University of Connecticut). Mutations were made in TRIO-9 cDNA using either overlap-extension PCR followed by In-fusion cloning (Clontech) or by Genscript™. TRIO-9 cDNAs were cloned into a pCAGGs vector containing IRES-mCherry. For electrophysiology experiments, a pFUGW vector expressing only GFP was co-expressed with pCAGG-IRES-mCherry TRIO-9 mutant constructs to enhance identification of transfected neurons. For co-immunoprecipitation and western blotting, N-terminus GFP-TRIO fusion constructs were generated with GFP inserted 3’ of the CDS within a pCAGGS vector. Similarly 1xFLAG sequence was inserted upstream of human *RAC1* sequence in the pCAGGS vector containing IRES-mCherry. GFP and mCherry expression were respectively used to confirm plasmid expression. For FLIM-FRET experiments, untagged TRIO constructs within the pCAGGS vector and the previously described Rac1 FRET-sensor within a pTrixEx-HisMyc backbone [31] were synthesized at Genscript™. All plasmids were confirmed by DNA sequencing.

### Electrophysiology

P6 to P8 Sprague Dawley rats of both sexes were used to prepare organotypic hippocampal slice cultures as previously described [32–34]. Tissue was isolated and a MX-TS tissue slicer (Siskiyou) was used to make 400 μm transverse sections. Tissue slices were placed on squares of Biopore Membrane Filter Roll (Millipore) and placed on Millicell Cell Culture inserts (Millipore) in 35 mm dishes. The slices were fed on alternate days with 1 ml of culture media (Invitrogen MEM + HEPES; catalog# 12360–038, Thermo Fisher Scientific; 25% horse serum (catalog# CCFAW001-148R02, UCSF Cell Culture Facility); HBSS (25%); and l-glutamine 1 mm).

Sparse biolistic transfections were performed on day in vitro 1 (DIV1) as previously described [35, 36]. Recordings were made on DIV7 or DIV9 in slice cultures on an upright Olympus BX50WI Microscope and perfused at 2.5 ml min^− 1^ with artificial CSF (aCSF) containing 119 mm NaCl, 2.5 mm KCl, 1 mm NaH_2_PO_4_, 26.2 mm NaHCO_3_, 11 mm glucose, 4 mm CaCl_2_, and 4 mm MgSO_4_ adjusted to osmolality of 305–315 mOsm, supplemented with 5 µm 2-chloroadenosine to dampen epileptiform activity and 0.1 mm picrotoxin to block GABA_A_ receptors. Borosilicate recording electrodes were filled with an internal solution containing 135 mm CsMeSO_4_, 8 mm NaCl, 10 mm HEPES, 0.3 mm EGTA, 5 mm QX-314, 4 mm Mg-ATP, and 0.3 mm Na-GTP adjusted to pH 7.3–7.4 and osmolarity of 290–295 mOsm. The aCSF was bubbled with 95% (v/v) O_2_ and 5% (v/v) CO_2_ to maintain pH.

Untransfected CA1 pyramidal neurons were identified using differential interference phase-contrast microscopy, while GFP-expressing CA1 pyramidal neurons cells were identified using epifluorescence microscopy. Postsynaptic currents were elicited by stimulation of stratum radiatum afferents with a monopolar glass electrode. AMPAR-evoked EPSCs (eEPSCs) were recorded by holding membrane voltage at − 70 mV, and measured from the same paired recording. No more than one pair was recorded from a single hippocampal slice. Membrane holding current, pipette series resistance, and input resistance were monitored throughout recording sessions. Data were gathered through a MultiClamp 700B amplifier (Molecular Devices), filtered at 2 kHz, and digitized at 10 kHz.

### Co-immunoprecipitation and immunoblotting

HEK293T cells were seeded in 100 mm culture dishes with DMEM containing 10% FBS, 1xGlutaMAX (Gibco, catalog#3505061), 100 U/ml penicillin and 100 µg/ml streptomycin at 37 °C and 5% CO2. Cells at 60–70% confluence were transfected with 5 µg 1xFLAG RAC1 and 5ug of either WT or mutant GFP-TRIO plasmid along with Lipofectamine 2000 (Thermo Fisher Scientific, catalog# 11668027) per the manufacturer’s protocol. 30 h later cells were washed with PBS and lysed in 1 mL of IP Lysis Buffer (Thermo Fisher Scientific, catalog# 87787) including cOmplete™ EDTA-free protease cocktail (Roche Diagnostics, catalog# 11873580001) in a QIAGEN TissueLyser II (for 3 min at 30 Hz). Samples were centrifuged at 20,000xg for 10 min at 4 °C. Supernatants containing equal amounts of total protein were incubated with GFP-trap magnetic beads (Chromotek, catalog# gtma) for 1 h at 4 °C. Beads were washed thrice with 1xPBS, 0.05% Tween20 and eluted with NuPAGE LDS sample buffer (Invitrogen) and NuPAGE Sample Reducing Agent (Invitrogen).

For immunoblotting experiments, samples were electrophoresed with SDS-PAGE gels (Invitrogen) and transferred to nitrocellulose membranes (Bio-Rad). Membranes were blocked with 5% bovine serum albumin in 1xTBST (10 mM Tris, pH 8.0, 150 mM NaCl, 0.1% Tween20) and incubated with primary antibodies diluted in blocking buffer at 4 °C overnight. Eluents were analyzed by immunoblotting with anti-GFP (Abcam, catalog# ab290), anti-FLAG (Proteintech, catalog# 20543-1-AP) and anti-GAPDH (CST, catalog# 2118) antibodies. All antibodies were used in a 1:1000 dilution. Blots were washed thrice with 1xTBST Buffer for 10 min at room temperature and incubated with IRDye® 800CW Donkey anti-Rabbit IgG antibodies (Li-cor). Blots were imaged using the Odyssey DLx (Li-cor).

### Fluorescence lifetime imaging (FLIM)

HEK293 cells were seeded on 35 mm glass-bottom dishes (Mattek, cat#P35G-1.5-14-C) precoated with Matrigel (Corning, cat# 356234) and cultured in DMEM containing 10% FBS, 1xGlutaMAX (Gibco, cat# 35050061), 100 U/ml penicillin and 100 µg/ml streptomycin at 37 °C and 5% CO2. 24 h later cells at 60–70% confluence were transfected with the 0.5 µg each Rac1 FRET-sensor and TRIO expression constructs using FuGENE HD Transfection Reagent (Promega).

16–20 h after transfection, cell culture media was replaced with phenol red-free 1xHBSS (Gibco, cat# 14-025-092) and cells were imaged on a Leica SP8 FALCON FLIM microscope. Laser excitation was delivered using a Spectra Physics Mai Tai DeepSee set to 840 nm to match peak excitation 2-photon excitation for Cerulean. Hybrid detectors were set to capture bandwidths of 450–500 nm and 518–558 nm to capture Cerulean and mVenus emission wavelengths respectively. Acquisition was performed using a 25x Fluotar L 25x/0.95NA water-immersion objective at 256 × 256 pixel resolution for maximal photons/pixel, 15 µs dwell time, 20 frame accumulations, and an overall acquisition time of approximately 30 s per frame.

### Experimental design and data analysis

For all experiments, at least 3 male and female rat pups were used. To test the effect of mutations on TRIO function, we employed an overexpression strategy instead of molecular replacement, because this would allow us to resolve dominant negative loss-of-function effects like the E1299W mutation, which would be significantly difficult to resolve with endogenous TRIO-9 depleted. Electrophysiological data are expressed as mean ± standard error measurement (SEM). Statistical significance for paired dual whole-cell patch clamp data was determined using Wilcoxon signed-rank test. Sample sizes in the present study are as reported previously (Herring and Nicoll 2016; Incontro et al. 2018). Data were analyzed and plotted in Microsoft Excel. For co-immunoprecipitation experiments, transfection, lysis and co-immunoprecipitation were performed for all conditions in parallel and immunoprecipitated lysates were loaded on the same SDS-PAGE gel. Images in figures are representative of four experimental and more than four technical repeats of TRIO-WT and all TRIO-9 mutants. For quantification of co-immunoprecipitation experiments, proteins were transferred to the same nitrocellulose membrane and probed together for technical consistency. Background adjusted band intensities were measured on Image Lab (Bio-Rad), graphed on Graph Pad Prism and analyzed for statistical significance using the Mann-Whitney test. Four replicates were quantified for each condition.

For FLIM experiments, images were analyzed using Leica’s FLIM software. Only the Cerulean (donor) channel was used for FLIM analysis. Dead cells in the field of view were removed using a region of interest and the entire remaining image was analyzed using exponential decay fitting and phasor plot analysis. Fluorescence decay curves were fitted with a 2-component exponential decay and the intensity-weighted mean fluorescence lifetime was extracted for each image. Approximately 20 images were collected for each condition and each condition was analyzed in pairwise comparisons using the Mann-Whitney test. The phasor plots were generated by combining data from all images of each mutant into a single phasor plot. Both co-immunoprecipitation and FLIM data were graphed as mean ± standard error of measurement (SEM) and analyzed on on GraphPad Prism. All p-values were denoted as follows: * for *p* < 0.05, ** for *p* < 0.01, *** for *p* < 0.001 and **** for *p* < 0.0001. All error bars represent SEM.

## Results

### Structure-based modeling predicts mutations disruptive for TRIO stability and RAC1 binding

A structure-based approach was used to predict mutations compromising TRIO’s interface in complex with RAC1 and mutations destabilizing the TRIO-GEF1 domain. The structure of TRIO’s GEF1 domain in complex with RAC1 has been solved [37], which allows accurate conformational modeling and evaluation of a mutation’s effects on TRIO GEF1 domain stability and RAC1 binding. We performed a comprehensive screen within the TRIO-GEF1 domain and selected four mutations predicted to reduce binding to RAC1 (Table 1) and four mutations predicted to reduce stability of the domain (Table 2). Predicted values of ∆∆G_*binding*_ or ∆∆G_*stability*_ were used as the primary criteria for mutation selection. Priority was given to the mutations with high values (in bold) for one of these properties (∆∆G_*stability*_ or ∆∆G_*binding*_) and values below threshold (< 3) for the other property (Tables 1 and 2). To diversify selection, we considered and sampled mutations located in distinct subregions of the GEF1 domain. All the mutation positions tested in our previous study were excluded from consideration [20]. In addition, two GEF1 domain mutations from gnomAD database were predicted to have negligible impact on TRIO stability and RAC1 binding and were used as control benign mutations (Tables 1 and 2). All mutations reported in this study have codon substitution probabilities *P* > 0.0003 [38]. Interestingly, our examination of the mutation E1299W, which has the lowest codon probability (*P* = 0.00037), identified this residue as critical for TRIO function. E1299K was recently discovered as a *de novo* mutation in a patient diagnosed with neurodevelopmental delays [22], highlighting the utility of our approach in predicting the pathogenicity of mutations of specific residues.

**Table 1:** Variants predicted to compromise TRIO-RAC1 binding interface (NCBI Reference Sequence for Trio: NM_007118.4)

Prediction	Variant	Base replacement	Effect on binding	∆∆G_stability_ (kCal/mol)	∆∆G_binding_ (kCal/mol)	Probability of variant [38]
Deleterious	E1299W	**GA**G→**TG**G	Disrupts H-bond with Y32 and T35 and V36 backbone	0.46	**4.24**	0.00037
Deleterious	C1387W	TG**C**→TG**G**	Bulky residue instead of small polar on the interaction interface	−1.71	**16.22**	0.00388
Deleterious	T1430W	**AC**G→**TG**G	Bulky residue instead of small polar on the interaction interface	−1.06	**6.82**	0.00076
Deleterious	A1464W	**GCC**→**TGG**	Bulky residue instead of small hydrophobic on the interaction interface	−0.12	**14.12**	0.00041
Benign	T1394A	**A**CT→**G**CT	TRIO-RAC1 binding interface, DH1 domain	0.55	1.90	0.03483

**Table 2:** Variants predicted to compromise TRIO-RAC1 stability (NCBI Reference Sequence for Trio: NM_007118.4)

Prediction	Variant	Base replacement	Effect on stability	∆∆G_stability_ (kCal/mol)	∆∆G_binding_ (kCal/mol)	Probability of variant [38]
Deleterious	E1304G	G**A**A→G**G**A	Disrupts H-bonds to R1428 and H1351, Y1432	**3.92**	1.00	0.01115
Deleterious	Y1318G	**TA**C→**GG**C	Distorts a-helix conformation, Disrupts H-bond to N1416 backbone	**5.46**	1.02	0.00094
Deleterious	Y1383A	**TA**T→**GC**T	Disrupts H-bonds to H1351 and Y1307	**3.83**	0.98	0.00163
Deleterious	G1453W	**G**G**C**→**T**G**G**	Small to big in hydrophobic core	**3.94**	4.57	0.00065
Benign	S1403F	T**C**C→T**T**C	TRIO surface, DH1 domain	1.45	0.97	0.00750

### Mutations predicted to compromise TRIO-RAC1 binding disrupt TRIO-9’s influence on glutamatergic synapse function

**Fig. 1:**
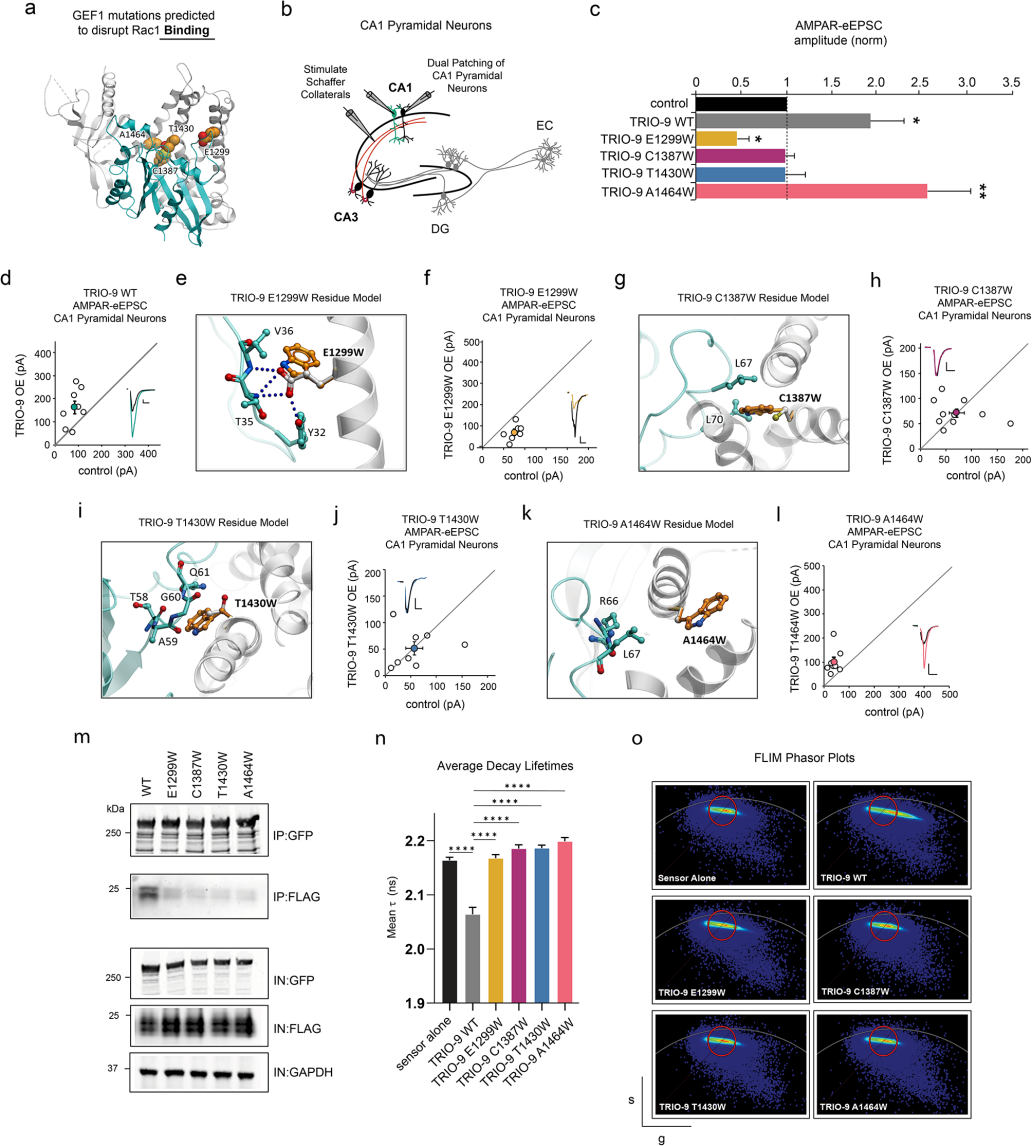
Mutations predicted to compromise TRIO-RAC1 binding disrupt TRIO-9’s influence on glutamatergic synapse function. **a** GEF1 mutations predicted to disrupt Rac1 binding. TRIO-9 is shown in grey, RAC1 is shown in cyan. **b** Electrophysiological recording setup. **c** Average AMPAR eEPSC amplitudes (± SEM) of neurons expressing wild-type (WT) TRIO-9, TRIO-9 E1299W, TRIO-9 C1387W, TRIO-9 T1430W and TRIO-9 A1464W normalized to their respective average control AMPAR-eEPSC amplitudes. Wilcoxon signed-rank was used to compare related samples (* = *p* < 0.05). **d**, **f**, **h**, **j**, **l** Scatterplots show AMPAR-eEPSC amplitudes for single pairs of control and transfected neurons (open circles). Filled circles show mean ± SEM. (Insets) Current traces from control (black) and transfected (various colors) neurons (Scale bars: 20 ms, 20 pA). **d** TRIO-9 expression increased AMPAR-eEPSC amplitude (*n* = 8 pairs, *p* < 0.05, Wilcoxon signed-rank test). **e**, **g**, **i**, **k** TRIO protein is shown in grey, RAC1 is shown in cyan. Mutated residue and close contacts are shown in sticks, with TRIO residues in grey, RAC1 residues in cyan, and mutation in orange. Hydrogen bonds are shown as blue dotted lines. **e** Interactions of E1299 amino acid residue in WT and mutant protein. **f** TRIO-9 E1299W expression showed a significant reduction in AMPAR-eEPSC amplitude (*n* = 7 pairs, *p* < 0.05, Wilcoxon signed-rank test), **g** Interactions of C1387 amino acid residue in WT and mutant protein. **h** TRIO-9 C1387W expression showed no increase in AMPAR-eEPSC amplitude (*n* = 9 pairs, *p* < 0.05, Wilcoxon signed-rank test), **i** Interactions of T1430 amino acid residue in WT and mutant protein j TRIO-9 T1430W expression showed no increase in AMPAR-eEPSC amplitude (*n* = 7 pairs, *p* < 0.05, Wilcoxon signed-rank test), **k** Interactions of A1464 amino acid residue in WT and mutant protein. **l** TRIO-9 A1464W expression showed an increase in AMPAR-eEPSC amplitude (*n* = 7 pairs, *p* < 0.05, Wilcoxon signed-rank test), **m** Representative immunoblots of lysates from HEK293T cells expressing GFP-TRIO-9 WT and GFP-TRIO-9 binding mutants each co-immunoprecipitated with FLAG-RAC1, probed with anti-GFP, anti-FLAG and anti-GAPDH, IN denotes input fractions, IP denotes immunoprecipitated fraction. **n** Mean Fluorescence Lifetime (τ) (± SEM) from exponential decay fit of field of ~20 cells per condition. Lower values indicate higher FRET and increase in RAC1 activity. Two-tailed Mann Whitney test was used to compare TRIO WT against sensor alone and each mutant against TRIO WT (*n* = 10 per condition, 2 technical replicates, ***p < 0.0001) **o** Phasor plots of flurorescence lifetime data for sensor alone, TRIO-9 WT and binding mutants. The x- and y- axes respectively display g = cosθ and s = sinθ transforms of the decay lifetime curve per pixel. The red circle was positioned around the ‘sensor alone’ signal and maintained in this position for all phasor plots. Extension to the right indicates an increase in FRET above the ‘sensor alone’ condition corresponding to a decrease in donor decay lifetime

Structure-based modeling predicted four mutations that would reduce free energy of TRIO binding to RAC1 (Table 1; Fig. 1a). Mutation E1299W was predicted to reduce TRIO-RAC1 binding by disruption of the comprehensive H-bond system between TRIO’s GEF1 domain and V36, T35, Y32 residues of RAC1 protein (Fig. 1e). Three other mutations (C1387W, T1430W, and A1464W) were predicted to interfere with TRIO-RAC1 binding by introducing steric clashes on the interaction interface. Specifically, mutation C1387W clashes with the sidechain of RAC1’s L70 residue (Fig. 1g), mutation T1430W clashes with the loop formed by residues A59, G60, and Q61 (Fig. 1i); mutation A1464W introduces steric clashes in the region of R66 and L67 residues of RAC1 (Fig. 1k). In summary, our computational model produced four mutants predicted to have a damaging effect on TRIO-RAC1 binding; three with the presence of a bulky residue instead of a small polar residue on the interaction surface (C1387W, A1464W and T1430W) and one mutation that disrupts an H-bond (E1299W) (Fig. 1a, e, g, i, k).

To determine whether these mutations predicted to disrupt TRIO-RAC1 binding would impact TRIO function, we designed individual TRIO-9 mutant expression constructs, which harbored mutations identified by our structure-based modeling. Since *TRIO-9* is the predominant TRIO isoform found in the brain we individually expressed WT TRIO-9 and TRIO-9 mutants in CA1 pyramidal cells in organotypic hippocampal slice cultures using biolistic transfection in organotypic slices [39]. To assess whether these mutations compromise TRIO-9’s influence on glutamatergic synapses, simultaneous whole-cell voltage clamp recordings of AMPA receptor-evoked excitatory postsynaptic currents (AMPAR-eEPSCs) were made from GFP-transfected CA1 pyramidal neurons and neighboring untransfected control neurons during Schaffer-collateral stimulation (Fig. 1b). This approach allows for a pair-wise, internally controlled comparison of the consequences from genetic manipulations in an intact tissue preparation [40–42]. As shown previously we find that expression of WT TRIO-9 results in a ∼2-fold increase in AMPAR-eEPSC amplitude (Fig. 1c, d, *p* = 0.03, *n* = 8, Wilcoxon-signed rank test) [40]. In marked contrast and as our modeling predicted, TRIO-9 mutants E1299W, C1387W and T1430W failed to produce increases in synaptic AMPAR-eEPSC amplitude relative to neighboring control neurons (Fig. 1c, f, h, j; for C11387W *p* = 0.76, *n* = 9; for T1430W *p* = 1, *n* = 8, Wilcoxon-signed rank test). Interestingly, TRIO-9 E1299W expression in neurons resulted in a marked decrease in AMPAR-eEPSC amplitude (Fig. 1c, f, *p* = 0.03, *n* = 7, Wilcoxon-signed rank test). This is indicative of a severe reduction in TRIO-9 GEF1 activity resulting in a dominant negative effect on neurotransmission. In contrast, TRIO-9 A1464W expression produced a significant increase in AMPAR-eEPSC amplitude that was comparable to WT TRIO-9 (Fig. 1c, l, *p* = 0.007, *n* = 8, Wilcoxon-signed rank test). Thus, our physiological approach established that 3 of 4 predictions were correct. We validated these findings using two orthogonal approaches: co-immunoprecipitation and immunoblotting to assay direct binding to RAC1 and FLIM-FRET to measure RAC1 activity. First, we expressed N-terminus GFP tagged WT Trio-9 or a binding mutant in HEK293T cells along with FLAG RAC1. We established that TRIO mutants are expressed at levels similar to WT TRIO-9 (Fig. 1m) and then probed FLAG RAC1 in each condition. We find that the binding mutants exhibit diminished TRIO-RAC1 binding (Fig. 1m and Additional file 1: Supplementary Fig. 2). The A1464W mutant also exhibits diminished binding in this assay, similar to the other three binding mutants. Co-immunoprecipitation data are quantified in Supplementary Fig. 1. To test the effect of each TRIO mutant on RAC1 activity, a Rac1 FRET-sensor was co-expressed with each TRIO construct in HEK293 cells. The previously reported Cerulean-mVenus (donor-acceptor) Rac1 FRET sensor measures RAC1 activity as function of donor-acceptor FRET [31]. In this paradigm, we measured donor decay-lifetime and found the Rac1 FRET-sensor displays a reduction in fluorescence lifetime relative to sensor alone when co-expressed with TRIO WT, indicating an increase RAC1 activity (Fig. 1n, o, *p* < 0.0001, *n* = 20, two-tailed Mann-Whitney test). In contrast, the binding mutants display longer decay lifetimes relative to the condition where TRIO-9 WT was expressed, indicating loss RAC1 activity (Fig. 1n, o, *p* < 0.0001, *n* = 20 for each binding mutants, two-tailed Mann-Whitney test). TRIO-9 A1464W also exhibits this phenotype and does not corroborate results from our electrophysiological data, and we outline possible explanations for this in the discussion. Overall, these data establish that our electrophysiological and orthogonal validation methods, along with the computational model can reliably predict and demonstrate the effect of mutations that are predicted to disrupt the binding of TRIO-9 to RAC1 at glutamatergic synapses.

### Mutations predicted to compromise TRIO-GEF1 stability disrupt TRIO-9’s influence on glutamatergic synapse function

**Fig. 2:**
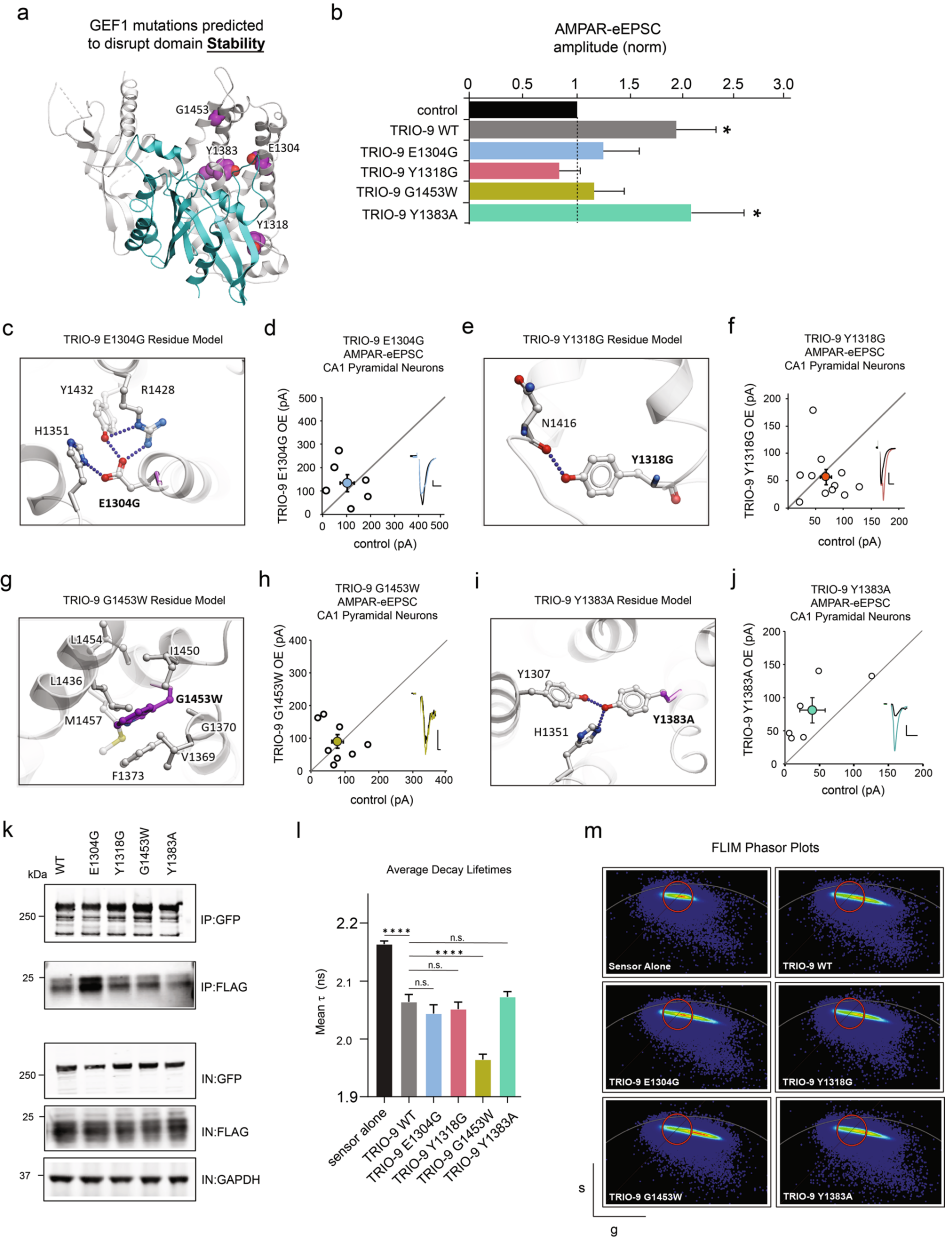
Mutations predicted to compromise TRIO-RAC1 stability disrupt TRIO-9’s influence on glutamatergic synapse function. **a** GEF1 mutations predicted to disrupt domain stability. TRIO protein shown in grey, RAC1 shown in cyan. **b** Average AMPAR-eEPSC amplitudes (± SEM) of neurons expressing WT TRIO-9 Y1318G, TRIO-9 E1304G, TRIO-9 G1453W and TRIO-9 Y1383A normalized to respective average control AMPAR-eEPSC amplitudes. Wilcoxon signed-rank test was used to compare related samples (*p* < 0.05). **d**, **f**, **h**, **j** Scatterplots show AMPAR-eEPSC amplitudes for pairs of control and transfected neurons (open circles). Filled circles show mean ± SEM. (Insets) Current traces from control (black) and transfected (various colors) neurons (Scale bars: 20 ms, 20 pA). **c**, **e**, **g**, **i** TRIO protein shown in grey, RAC1 shown in cyan. Mutated residue and close contacts are shown in sticks, with TRIO residues in grey, RAC1 residues in cyan, and mutations in magenta. Blue dotted lines indicate Hydrogen bonds. **c** Interactions of E1304 amino acid residue in WT and mutant protein. **d** TRIO-9 E1304G expression showed no increase in AMPAR-eEPSC amplitude (*n* = 6 pairs, *p* < 0.05, Wilcoxon signed-rank rest), **e** Interactions of Y1318 amino acid residue in WT and mutant protein. **f** TRIO-9 Y1318G expression showed no increase in AMPAR-eEPSC amplitude (*n* = 11 pairs, *p* < 0.05, Wilcoxon signed-rank test), **g** Interactions of G1453 amino acid residue in WT and mutant protein. **h** TRIO-9 G1453W expression showed no increase in AMPAR-eEPSC amplitude (*n* = 8 pairs, *p* < 0.05, Wilcoxon signed-rank test), **i** Interactions of Y1383 amino acid residue in WT and mutant protein. **j** TRIO-9 Y1383A expression showed an increase in AMPAR-eEPSC amplitude (*n* = 6 pairs, *p* < 0.05, Wilcoxon signed-rank test) **k** Representative immunoblots of lysates from HEK293T cells expressing GFP-TRIO-9 WT and GFP-TRIO-9 binding mutants each co-immunoprecipitated with FLAG-RAC1, probed with anti-GFP, anti-FLAG and anti-GAPDH, IN denotes input fractions, IP denotes immunoprecipitated fraction. **l** Mean Fluorescence Lifetime (τ) (± SEM) from exponential decay fit of field of ~20 cells per condition. Lower values indicate higher FRET and increase in RAC1 activity. Two-tailed Mann Whitney test was used to compare TRIO WT against sensor alone and each mutant against TRIO WT (*n* = 20 per condition, 2 experimental replicates, ****p < 0.0001, two-tailed Mann Whitney test) **m** Phasor plots of flurorescence lifetime data for sensor alone, TRIO-9 WT and binding mutants. The x- and y- axes respectively display g = cosθ and s = sinθ transforms of the decay lifetime curve per pixel. The red circle was positioned around the ‘sensor alone’ signal and maintained in this position for all phasor plots. Extension to the right indicates an increase in FRET above the ‘sensor alone’ condition corresponding to a decrease in donor decay lifetime

In addition to mutations that disrupt binding, our structure-based computational predictions identified mutants that would disrupt the stability of the TRIO GEF1-RAC1 binding site. Our model predicted three mutations (E1304G, Y1318G, and Y1383A) to reduce GEF1 domain stability by interrupting the network of internal hydrogen bonds (Table 2; Fig. 2a). Mutation E1304G prevents formation of an extensive H-bond network between the sidechain of four residues, E1304, H1351, Y1432, and R1428 (Fig. 2c). Mutation Y1318G destroys the H-bond interaction with the backbone of N1416 residue (Fig. 2e). Mutation Y1383A abolishes the H-bond interactions with the side chains of Y1307 and H1351 (Fig. 2i). The fourth mutation, G1453W, leads to the destabilization of the TRIO’s GEF1 domain by introducing internal clashes between α-helices, specifically with residues L1436 and F1373 (Fig. 2g). In summary, the predicted mutations affect binding by disrupting H-bonds to specific residues (TRIO E1304G and Y1383A), distort the α-helix conformation and a backbone H-bond (TRIO Y1318G), and harbor a modification resulting in a larger hydrophobic core (TRIO G1453W) (Fig. 2a, c, e, g, i).

To determine the effect these mutations have on TRIO-9 function, we designed individual TRIO-9 mutant expression constructs, each of which harbored a mutation predicted to disrupt the stability of TRIO-9’s GEF1 domain. By recording post-synaptic currents as described previously, we found that three out of four TRIO-9 mutants we tested showed a pronounced lack of increase in AMPAR-eEPSC amplitude relative to WT TRIO-9 (Fig. 2b). Specifically, TRIO-9 E1304G, Y1318G and G1453W mutations prevented TRIO-9 mediated potentiation of synapses (Fig. 2d, f, h; for E1304G *p* = 0.69, *n* = 6; for Y1318G *p* = 0.32, *n* = 11, for G1453W *p* = 0.84, *n* = 8; Wilcoxon-signed rank test). This finding is consistent with the predictions from our structure-based computational model. A significant effect was not observed on AMPAR-current amplitudes for all mutants except TRIO-9 Y1383A, which increased AMPAR-eEPSC amplitude similar to WT TRIO-9 (Fig. 2j, *p* = 0.3, *n* = 6, Wilcoxon-signed rank test). Taken together, our combined structure-based and electrophysiological approaches confirm the impact of mutations predicted to alter GEF1-domain stability on glutamatergic synapse function.

In co-immunoprecipitation experiments, where Trio-9 mutant expression constructs were co-expressed with FLAG RAC1 in HEK293T cells, we find that the stability mutants do not display a loss in RAC1 binding relative to Trio-9 WT (Fig. 2k and Additional file 1: Supplementary Fig. 3). We observed a similar lack of effect from these mutations in FLIM-FRET experiments, which displayed Rac1 FRET-sensor decay lifetimes indistinguishable from TRIO-9 WT (Fig. 2l, m, for E1304G *p* = 0.31, for Y1318G *p* = 0.31, for Y1383A *p* = 0.49, two-tailed Mann-Whitney test). TRIO-9 G1453W was the exception, and induced lower sensor decay lifetime relative to TRIO-9 WT indicating an increase in RAC1 activity (Fig. 2l, m, *p* < 0.0001, two-tailed Mann-Whitney test). Altogether, we find that for mutations predicted to affect TRIO-9 conformational stability, results from orthogonal approaches do not correlate with the robust effects observed in our electrophysiological data from neurons in an intact circuit preparation. We address possible caveats and limitations of these approaches involving heterologous cell-expression systems (see Discussion).

### Mutations predicted to be benign to TRIO-RAC1 interaction do not interfere with TRIO-9’s effect on glutamatergic synapse function

**Fig. 3:**
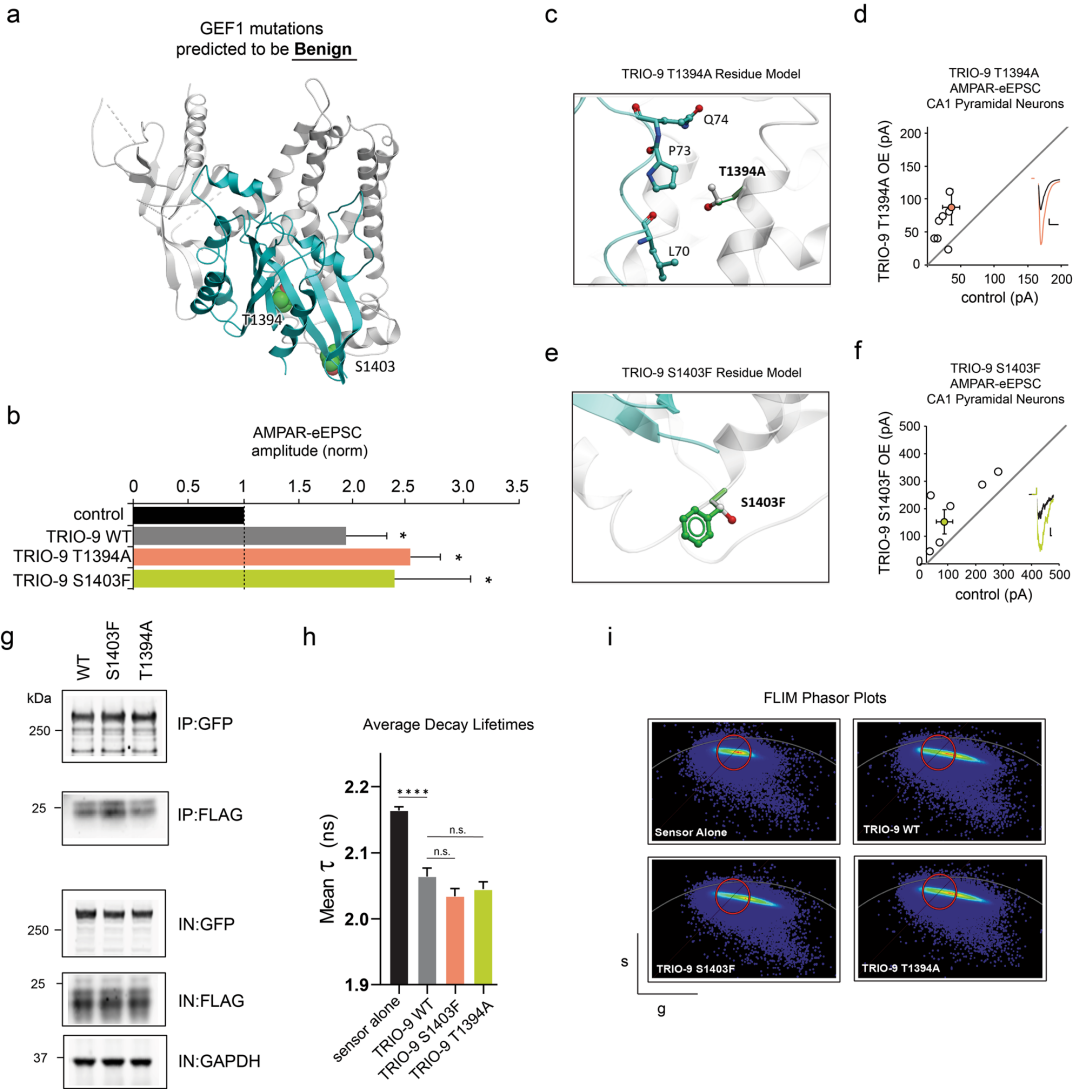
Mutations predicted to be benign to TRIO-RAC1 interaction do not interfere with TRIO-9’s influence on glutamatergic synapse function. **a** GEF1 mutations predicted to be benign **b** Average AMPAR-eEPSC amplitudes (± SEM) of neurons expressing WT TRIO-9, TRIO-9 Y1394A and TRIO-9 T1394A normalized to their respective average control AMPAR-eEPSC amplitudes. Wilcoxon Rank Sum Test was used to compare related samples (* = *p* < 0.05). **c, e** In Residue Models, TRIO protein shown in grey, RAC1 shown in cyan. Mutated residues and close contacts shown in sticks, with TRIO residues in grey and mutation in green. **c** Interactions of T1394 amino acid residue in WT and mutant protein. **d, f** Scatterplots show AMPAR-eEPSC amplitudes for single pairs of control and transfected neurons (open circles). Filled circles show mean ± SEM. (Insets) Current traces from control (black) and transfected (various colors) neurons (Scale bars: 20 ms, 20 pA). **d** TRIO-9 T1394A expression increased AMPAR-eEPSC amplitude (*n* = 7 pairs, *p* < 0.05, Wilcoxon signed-rank test), **e** Interactions of S1403 amino acid residue in WT and mutant protein. **f** TRIO-9 S1403F expression increased AMPAR-eEPSC amplitude (*n* = 7 pairs, *p* < 0.05, Wilcoxon signed-rank test) **g** Representative immunoblots of lysates from HEK293 cells expressing GFP-TRIO-9 WT and GFP-TRIO-9 binding mutants each co-immunoprecipitated with FLAG-RAC1, probed with anti-GFP, anti-FLAG and anti-GAPDH, IN denotes input fractions, IP denotes immunoprecipitated fraction. **h** Mean Fluorescence Lifetime (τ) (± SEM) from exponential decay fit of field of ~20 cells per condition. Lower values indicate higher FRET and increase in RAC1 acitvity. Two-tailed Mann Whitney test was used to compare TRIO WT against sensor alone and each mutant against TRIO WT (*n* = 10 per condition, 2 technical replicates, ****p < 0.0001, two-tailed Mann Whitney test) **i** Phasor plots of fluorescence lifetime data for sensor alone, TRIO-9 WT and binding mutants. The x- and y- axes respectively display g = cosθ and s = sinθ transforms of the decay lifetime curve per pixel. The red circle was positioned around the ‘sensor alone’ signal and maintained in this position for all phasor plots. Extension to the right indicates an increase in FRET above the ‘sensor alone’ condition corresponding to a decrease in donor decay lifetime

We identified mutations from the gnomAD database that are identified as missense mutations within the GEF1 domain but were predicted to be benign by our computational model. These mutations were T1394A, on the TRIO-RAC1 binding interface within the DH1 domain, and S1403F on the surface of TRIO’s DH1 domain (Fig. 3a, c, e). To determine whether our computational predictions were accurate, we biolistically transfected hippocampal slices with each TRIO-9 mutant. We recorded AMPAR-eEPSCs from CA1 pyramidal neurons while stimulating Schaffer-collateral afferents and found that both mutants produced average current amplitudes significantly larger than controls similar to the synaptic phenotype observed with WT TRIO-9 (Fig. 3b, d, f; for T1394A *p* = 0.02, *n* = 8; for S1403F *p* = 0.03, *n* = 6; Wilcoxon-signed rank test). These data demonstrate that these control mutations do not affect TRIO-9-mediated potentiation of AMPAR-eEPSCs.

We tested these control TRIO-9 mutations found to be benign in our electrophysiological assay, in co-immunoprecipitation experiments with FLAG RAC1 in HEK293T cells and found FLAG-RAC1 binding for both mutants comparable to TRIO-9 WT (Fig. 3g and Additional file 1: Supplementary Fig. 4). Similarly, in FLIM-FRET experiments, we find Rac1 FRET-sensor decay lifetimes in conditions with control Trio-9 mutants to be indistinguishable from TRIO-9 WT (Fig. 3h, i, for S1403F *p* = 0.082 and for T1394A *p* = 0.4, two-tailed Mann-Whitney test). Taken together, all three approaches confirm these control mutations do not effect RAC1 binding or activity.

## Discussion

With the increasing availability of postnatal human genome sequencing, there is a growing demand for accurate and robust computational tools predicting genomic mutations carrying increased risk of ASD or related neurodevelopmental disorders. Since individual *de novo* missense mutations are exceedingly rare, they are not identified by genome-wide association analysis (GWAS), but through other approaches like exome sequencing. Moreover, the impact of missense mutations on protein function is difficult to assess. While frameshift mutations, nonsense mutations and copy number variations (CNVs) in a gene are relatively easy to identify and assess as they eliminate key domains or the whole protein, identification of *de novo* missense mutations require analysis of sequence and their impact is difficult to ascertain. *In silico* prediction tools including PolyPhen2 and SIFT [43–47] have been developed to predict whether a missense mutation in a gene affects protein function. These tools rely heavily on comparative analysis of sequence variations, while using structural information only in the form of general descriptors of protein residues such as surface area and B-factor. However, these models are trained on variant frequency within primate and human sequence data to predict the effect of an amino acid substitution and do not account for effects on molecular interfaces or binding sites. While fast and useful, these *in silico* prediction tools are prone to false positives, often incorrectly classifying mutations as damaging or significantly overestimating the damaging effect of missense mutations [43, 44]. The recently published Alpha Missense method predicts variant pathogenicity based on Alpha Fold-derived protein structures and demonstrates greater accuracy models by taking 3D structural information into account [48]. We compared our predictive data to Alpha Missense and found both models display 80% accuracy for the mutants described in this study (Additional file 1: Supplementary Table 1).

Our approach is based on a detailed all-atom energy-based structural model of mutations to predict their effect on protein stability, interaction with a functional partner, and the biological function promoted by this interaction. In the case of the TRIO-RAC1 pathway, we use a high-resolution structure of TRIO’s GEF1 domain with RAC1 for modeling. Our previous study identified clustering of *de novo* ASD-associated mutations in the GEF/DH1 domain of TRIO, a region that binds directly to RAC1, and used this structure-based computational analysis to predict that these mutations would produce pathological disruptions in glutamatergic neurotransmission. We previously characterized the ASD-related *de novo* mutations within this hotspot and implicated the bi-directional alterations in neurotransmission produced by these mutations in ASD pathology. In the present study, we use this structure-based computational approach as a predictive tool to suggest potentially deleterious mutations in TRIO-9. To test the computational predictions experimentally, we used dual whole cell voltage-clamp in hippocampal slice cultures to assess the effect of these mutations in neurons. We show that 75% (6 out of 8) of mutations that were predicted as deleterious do indeed disrupt TRIO-RAC1 mediated glutamatergic synapse function. We also showed that mutations in TRIO predicted to disrupt TRIO-RAC1 binding display reduced binding of mutant TRIO protein when co-immunoprecipitated with RAC1 in a heterologous expression system. These data were supported by additional findings in FLIM-FRET assays that showed lower Rac1 FRET-sensor activity in TRIO binding mutants relative to TRIO WT. However, the orthogonal assays in this study were performed in cell lines, which do not recapitulate the synaptic proteome and associated mechanisms of proteostasis that regulate protein folding and subcellular compartment-specific functions in neurons [49, 50]. It is possible specific molecular chaperones and co-factor proteins present at neuronal synaptic compartments are required for the proper expression and stability of TRIO, and canonical TRIO-RAC1 interaction. Additionally, we do not assess the expression or localization of these mutants in our neuronal assays or directly measure the effect of destabilizing mutants on GEF1 domain stability. Any of these factors could explain why the stability mutations and binding mutation A1464W do not phenocopy the effects observed in neuronal electrophysiological experiments performed in the context of an intact synaptic circuit. High-throughput ligand binding or variant expression screens to assay the functional effect of TRIO variants on expression, stability or catalytic activity could prove a promising area of research [51–53].

Employing direct structural modeling makes it possible to distinguish between disrupting and benign mutations on the functional interface more accurately compared to using just general structural descriptors, and thus overcome the high false-positive rate of sequence-based approaches. A comparison of our results with PolyPhen2 predictions (Additional file 1: Supplementary Table 1) shows that PolyPhen2 predicts two GEF1 mutations of healthy individuals from the gnomAD database as potentially deleterious. We show that both mutations predicted to be neutral by our approach, were experimentally validated as lacking any significant effect on TRIO-9 function. The fact that these mutations were predicted as potentially deleterious by PolyPhen2 supports the assertion that our structure-based approach reduces false positive predictions.

**Fig. 4:**
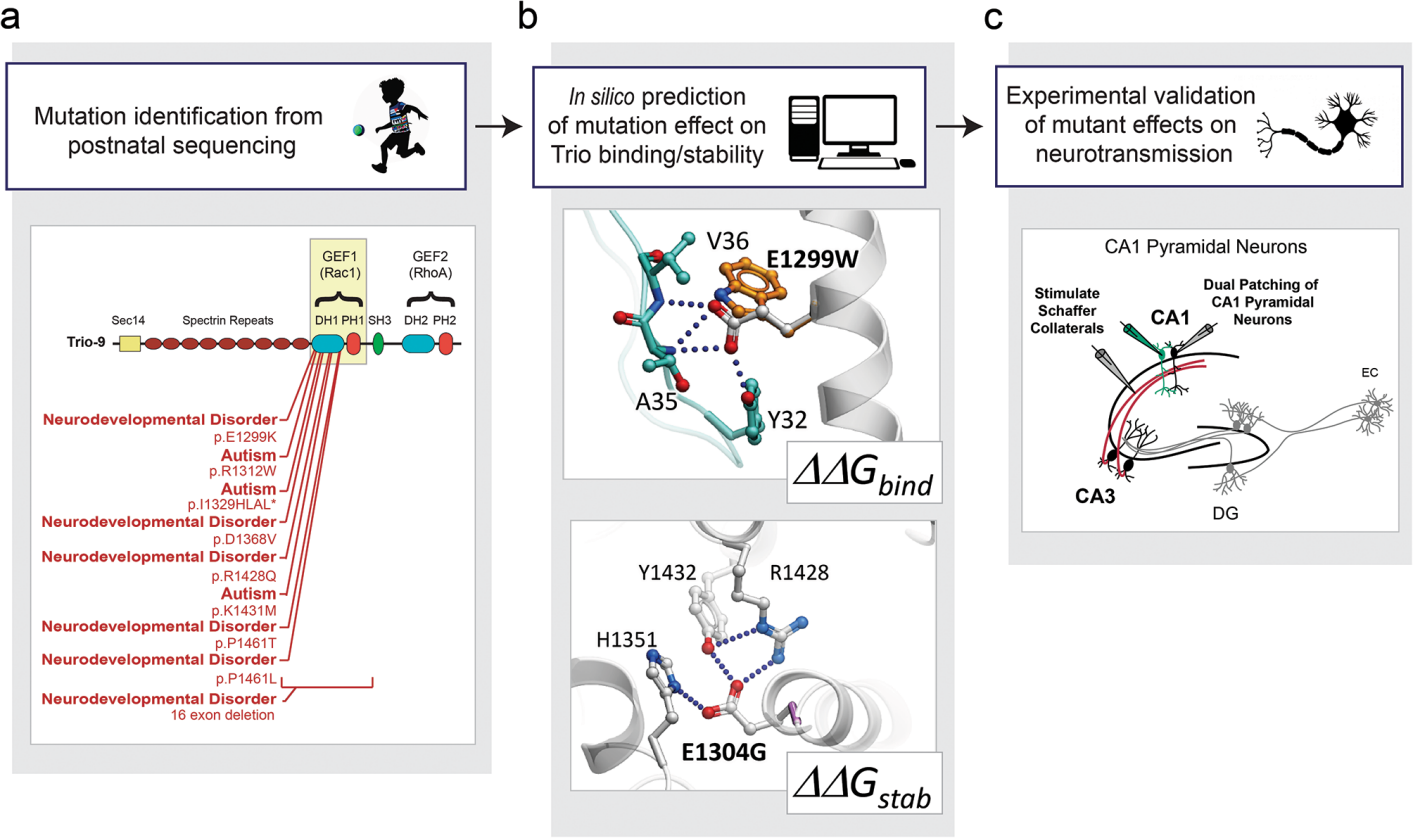
Workflow for structure-based prediction method to identify pathological ASD-related *de novo* mutations in TRIO’s DH1 domain in humans with ASD/ID. (**a**) Mutation identification from postnatal clinical gene sequencing. Example image below shows cluster of missense, nonsense and CNVs in TRIO, identified in individuals with ASD-related disorders. The different protein domains are indicated, starting with the N-terminus: Sect. 14 domain (dark green), Spectrin repeats (maroon), GEF1 domain composed of a Dbl homology domain (DH1, in orange) and a Pleckstrin homology domain (PH1, in pink), Src homology 3 domain (SH3) (green), and the GEF2 domain (composed of a Dbl homology domain (DH2, in blue) and a Pleckstrin homology domain (PH2, in grey). For each mutation, the individual’s diagnosis is given along with information about the alteration of TRIO’s amino acid sequence. Position of amino-acid mutations from NP_009049.2, (**b**) An *in silico* prediction from our structure-based method on whether a patient’s mutations impacts the free energy change in binding (∆∆G_*binding*_) or protein stability (∆∆G_*stability*_) of the TRIO-RAC1 complex. Representative mutations shown in images below are E1299W and E1304G predicted to impact protein binding (∆∆G_*binding*_) and protein stability (∆∆G_*stability*_) respectively. TRIO protein is shown in grey, RAC1 is shown in cyan. Mutated residues and close contacts are shown in sticks, with TRIO residues in grey, RAC1 residues in cyan, and mutations in orange or magenta. Hydrogen bonds are shown as blue dotted lines. (**c**) Electrophysiological recording setup shown in image. Experimental validation of the impact of the mutant TRIO variant on glutamatergic neurotransmission assessed using dual-whole cell voltage-clamp of paired CA1 pyramidal neurons

This incidence of false positives from existing prediction algorithms demonstrates a need for more sophisticated and accurate predictive approaches that account for protein structures and residue interactions on protein-protein interfaces. Advanced structure-based modeling as described here, could be used to analyze variants detected in genomic sequencing in the clinic. Combined with in vitro experimental validation, this could provide a reliable assessment of whether a given mutation would be detrimental to protein and synaptic function (Fig. 4). Such information will aid significantly in identifying those mutations in individuals that confer elevated disease risk. Ultimately, this may enable better identification and classification of syndromic forms of ASD/ID.

In the present study, our experimental validation has informed retrospective analysis of predictions from our model that could further improve the accuracy of the model. Out of four mutations predicted to affect TRIO-RAC1 binding, three were confirmed in vitro. Mutation E1299W, which shows a dominant-negative effect, stands out as one disrupting a comprehensive network of hydrogen bonds between TRIO and RAC1 proteins, in agreement with the high importance of polar interactions for selective binding. When we overexpress this mutant, it has a dominant negative effect on synaptic neurotransmission, possibly because it produces a greater reduction in enzymatic activity than other mutants and outcompetes endogenous TRIO activity. Interestingly, a mutation at this residue was reported in an individual diagnosed with moderate neurodevelopmental delays and an associated loss of RAC1 activity was biochemically confirmed [22]. Our unbiased model and screen independently identified the impact of a mutation at this residue, supporting the predictive power of our method. Furthermore, in our previous study, all ASD/ID-associated TRIO GEF1 mutations tested produced a similar dominant negative effect [20]. Thus, this phenotype may serve as a useful biomarker in identifying high-confidence ASD/ID-associated *TRIO* variants in humans. The effect of the other three mutations (C1387W, A1464W and T1430W) was predicted based on introducing steric clashes between the proteins. While C1387W and T1430W were indeed disruptive, mutation A1464W did not have a significant effect. Although the model predicted that the A1464W mutant would produce a steric clash interfering with the TRIO-RAC1 protein interaction, our retrospective analysis suggests that a minor backbone movement in this region might mitigate the steric hindrance and avoid the detrimental effect. Similarly, out of four mutations predicted to affect protein stability, the experimental data for the Y1383A mutant did not recapitulate our model’s prediction. Even though the mutation was predicted to disrupt polar interactions to two residues, this might be compensated by some backbone adjustment and more tight packing, preserving the protein stability. Thus, accurately taking into account some limited backbone adjustments might further improve the predictive power of the approach.

## Limitations

Our study provides a relatively simple and accurate method to determine whether missense mutations in TRIO’s GEF1 domain in individuals may compromise TRIO function and contribute to an increased risk of ASD/ID. While this new diagnostic tool represents a vast improvement over those previously available, the penetrance of missense mutations in TRIO’s GEF1 domain that compromise TRIO function is presently unknown. As such, missense mutations identified as detrimental to TRIO function in our method do not provide conclusive evidence that ASD/ID will develop in individuals who have yet to exhibit symptoms of such disorders. Additionally, even when mutations predicted as detrimental to TRIO function with our method are found present in individuals diagnosed with ASD/ID, there is no guarantee that such mutations are solely responsible for the disorder. As a greater number of individuals both with and without ASD/ID are identified that harbor missense mutations within TRIO’s GEF1 domain, estimations regarding the accuracy of our new method in predicting ASD/ID risk will become possible.

## Conclusions

The structure-based approach described here may be applied to other critical protein-interactions that are causally involved in ASD/ID. One key requirement for our structure-based approach is the availability of accurate structural models of the protein complexes. Currently, this field is being rapidly populated by new structural biology techniques like high-resolution cryo-Electron Microscopy [54, 55], X-ray Free Electron Laser XFEL [56], or hybrid structural modeling methods [57]. Advances in ab initio structure-determination methods like Alpha-Fold [58] may help to fill this gap, providing high-resolution structural information for a vast majority of proteins. This will facilitate the development of sophisticated structure-based approaches like ours, to identify pathological missense mutations more accurately in the human genome, which will lead to better diagnostic predictions for ASD.

### Electronic supplementary material

Below is the link to the electronic supplementary material.

**Additional file 1**: **Supplementary Table 1** Comparison of predictions from our model vs. Polyphen2, FATTHM and Alpha Missense. **Supplementary Fig. 1** Quantification of co-immunoprecipitation of TRIO mutants with RAC1. **Supplementary Fig. 2** Immunoblots of co-immunoprecipitation of TRIO binding mutants with RAC1. **Supplementary Fig. 3** Immunoblots of co-immunoprecipitation of TRIO stability mutants with RAC1. **Supplementary Fig. 4** Immunoblots of co-immunoprecipitation of TRIO benign mutants with RAC1

## Data Availability

No datasets were generated or analysed during the current study.

## References

[1] Baio J, Wiggins L, Christensen DL, Maenner MJ, Daniels J, Warren Z (2018). Prevalence of Autism Spectrum Disorder among children aged 8 years - Autism and Developmental Disabilities Monitoring Network, 11 sites, United States, 2014. MMWR Surveill Summ.

[2] Bai D, Yip BHK, Windham GC, Sourander A, Francis R, Yoffe R (2019). Association of Genetic and Environmental Factors with Autism in a 5-Country cohort. JAMA Psychiatry.

[3] Hansen SN, Schendel DE, Francis RW, Windham GC, Bresnahan M, Levine SZ (2019). Recurrence risk of Autism in siblings and cousins: a multinational, Population-based study. J Am Acad Child Adolesc Psychiatry.

[4] Rylaarsdam L, Guemez-Gamboa A (2019). Genetic causes and modifiers of Autism Spectrum Disorder. Front Cell Neurosci.

[5] Devlin B, Scherer SW (2012). Genetic architecture in autism spectrum disorder. Curr Opin Genet Dev.

[6] Veenstra-Vanderweele J, Christian SL, Cook EH (2004). Jr. Autism as a paradigmatic complex genetic disorder. Annu Rev Genomics Hum Genet.

[7] Grove J, Ripke S, Als TD, Mattheisen M, Walters RK, Won H (2019). Identification of common genetic risk variants for autism spectrum disorder. Nat Genet.

[8] Feliciano P, Zhou X, Astrovskaya I, Turner TN, Wang T, Brueggeman L (2019). Exome sequencing of 457 autism families recruited online provides evidence for autism risk genes. npj Genomic Med.

[9] Satterstrom FK, Kosmicki JA, Wang J, Breen MS, De Rubeis S, An J-Y (2020). Large-scale exome sequencing study implicates both developmental and functional changes in the Neurobiology of Autism. Cell.

[10] Stein JL, Parikshak NN, Geschwind DH (2013). Rare inherited variation in autism: beginning to see the forest and a few trees. Neuron.

[11] Volkmar F, Siegel M, Woodbury-Smith M, King B, McCracken J, State M (2014). Practice parameter for the Assessment and Treatment of children and adolescents with Autism Spectrum Disorder. J Am Acad Child Adolesc Psychiatry.

[12] Munnich A, Demily C, Frugere L, Duwime C, Malan V, Barcia G (2019). Impact of on-site clinical genetics consultations on diagnostic rate in children and young adults with autism spectrum disorder. Mol Autism.

[13] Du X, Gao X, Liu X, Shen L, Wang K, Fan Y (2018). Genetic diagnostic evaluation of Trio-based whole exome sequencing among children with diagnosed or suspected Autism Spectrum Disorder. Front Genet.

[14] Herman GE, Henninger N, Ratliff-Schaub K, Pastore M, Fitzgerald S, McBride KL (2007). Genetic testing in autism: how much is enough?. Genet Med.

[15] Abdul-Rahman OA, Hudgins L (2006). The diagnostic utility of a genetics evaluation in children with pervasive developmental disorders. Genet Med.

[16] Schaefer GB, Lutz RE (2006). Diagnostic yield in the clinical genetic evaluation of autism spectrum disorders. Genet Med.

[17] Barton KS, Tabor HK, Starks H, Garrison NA, Laurino M, Burke W (2018). Pathways from autism spectrum disorder diagnosis to genetic testing. Genet Medicine: Official J Am Coll Med Genet.

[18] Schaefer GB, Mendelsohn NJ, for the Professional P, Guidelines C (2013). Clinical genetics evaluation in identifying the etiology of autism spectrum disorders: 2013 guideline revisions. Genet Med.

[19] Volk L, Chiu S-L, Sharma K, Huganir RL (2015). Glutamate synapses in Human Cognitive disorders. Annu Rev Neurosci.

[20] Sadybekov A, Tian C, Arnesano C, Katritch V, Herring BE (2017). An autism spectrum disorder-related de novo mutation hotspot discovered in the GEF1 domain of Trio. Nat Commun.

[21] Bonnet M, Roche F, Fagotto-Kaufmann C, Gazdagh G, Truong I, Comunale F (2023). Pathogenic TRIO variants associated with neurodevelopmental disorders perturb the molecular regulation of TRIO and axon pathfinding in vivo. Mol Psychiatry.

[22] Barbosa S, Greville-Heygate S, Bonnet M, Godwin A, Fagotto-Kaufmann C, Kajava AV (2020). Opposite modulation of RAC1 by mutations in TRIO is Associated with distinct, Domain-Specific Neurodevelopmental disorders. Am J Hum Genet.

[23] Katrancha SM, Wu Y, Zhu M, Eipper BA, Koleske AJ, Mains RE (2017). Neurodevelopmental disease-associated de novo mutations and rare sequence variants affect TRIO GDP/GTP exchange factor activity. Hum Mol Genet.

[24] Blangy A, Vignal E, Schmidt S, Debant A, Gauthier-Rouvière C, Fort P (2000). TrioGEF1 controls rac- and Cdc42-dependent cell structures through the direct activation of rhoG. J Cell Sci.

[25] O’Roak BJ, Vives L, Girirajan S, Karakoc E, Krumm N, Coe BP (2012). Sporadic autism exomes reveal a highly interconnected protein network of de novo mutations. Nature.

[26] de Ligt J, Willemsen MH, van Bon BW, Kleefstra T, Yntema HG, Kroes T (2012). Diagnostic exome sequencing in persons with severe intellectual disability. N Engl J Med.

[27] Sanders SJ, Murtha MT, Gupta AR, Murdoch JD, Raubeson MJ, Willsey AJ (2012). De novo mutations revealed by whole-exome sequencing are strongly associated with autism. Nature.

[28] De Rubeis S, He X, Goldberg AP, Poultney CS, Samocha K, Cicek AE (2014). Synaptic, transcriptional and chromatin genes disrupted in autism. Nature.

[29] Karczewski KJ, Francioli LC, Tiao G, Cummings BB, Alföldi J, Wang Q (2020). The mutational constraint spectrum quantified from variation in 141,456 humans. Nature.

[30] Schapira M, Totrov M, Abagyan R (1999). Prediction of the binding energy for small molecules, peptides and proteins. J Mol Recognit.

[31] Timmerman I, Heemskerk N, Kroon J, Schaefer A, van Rijssel J, Hoogenboezem M (2015). A local VE-cadherin and Trio-based signaling complex stabilizes endothelial junctions through Rac1. J Cell Sci.

[32] Stoppini L, Buchs PA, Muller D (1991). A simple method for organotypic cultures of nervous tissue. J Neurosci Methods.

[33] Prang P, Del Turco D, Kapfhammer JP (2001). Regeneration of entorhinal fibers in mouse slice cultures is age dependent and can be stimulated by NT-4, GDNF, and modulators of G-proteins and protein kinase C. Exp Neurol.

[34] Bonnici B, Kapfhammer JP (2009). Modulators of signal transduction pathways can promote axonal regeneration in entorhino-hippocampal slice cultures. Eur J Pharmacol.

[35] Lu W, Shi Y, Jackson AC, Bjorgan K, During MJ, Sprengel R (2009). Subunit composition of synaptic AMPA receptors revealed by a single-cell genetic approach. Neuron.

[36] Schnell E, Sizemore M, Karimzadegan S, Chen L, Bredt DS, Nicoll RA (2002). Direct interactions between PSD-95 and stargazin control synaptic AMPA receptor number. Proc Natl Acad Sci U S A.

[37] Chhatriwala MK, Betts L, Worthylake DK, Sondek J (2007). The DH and PH domains of Trio Coordinately Engage rho GTPases for their efficient activation. J Mol Biol.

[38] Schneider A, Cannarozzi GM, Gonnet GH (2005). Empirical codon substitution matrix. BMC Bioinformatics.

[39] McPherson CE, Eipper BA, Mains RE (2005). Multiple novel isoforms of Trio are expressed in the developing rat brain. Gene.

[40] Herring BE, Nicoll RA. Kalirin and Trio proteins serve critical roles in excitatory synaptic transmission and LTP. Proceedings of the National Academy of Sciences. 2016;113(8):2264. 10.1073/pnas.1600179113 PMC4776457 26858404

[41] Rao S, Kay Y, Herring BE (2019). Tiam1 is critical for glutamatergic synapse structure and function in the Hippocampus. J Neurosci.

[42] Tian C, Kay Y, Sadybekov A, Rao S, Katritch V, Herring BE (2018). An intellectual disability-related missense mutation in Rac1 prevents LTP induction. Front Mol Neurosci.

[43] Zhao N, Han JG, Shyu CR, Korkin D (2014). Determining effects of non-synonymous SNPs on protein-protein interactions using supervised and semi-supervised learning. PLoS Comput Biol.

[44] Gnad F, Baucom A, Mukhyala K, Manning G, Zhang Z (2013). Assessment of computational methods for predicting the effects of missense mutations in human cancers. BMC Genomics.

[45] Khurana E, Fu Y, Chen J, Gerstein M (2013). Interpretation of genomic variants using a unified biological network approach. PLoS Comput Biol.

[46] Reva B, Antipin Y, Sander C (2011). Predicting the functional impact of protein mutations: application to cancer genomics. Nucleic Acids Res.

[47] Kumar P, Henikoff S, Ng PC (2009). Predicting the effects of coding non-synonymous variants on protein function using the SIFT algorithm. Nat Protoc.

[48] Cheng J, Novati G, Pan J, Bycroft C, Zemgulyte A, Applebaum T (2023). Accurate proteome-wide missense variant effect prediction with AlphaMissense. Science.

[49] Shemesh N, Jubran J, Dror S, Simonovsky E, Basha O, Argov C (2021). The landscape of molecular chaperones across human tissues reveals a layered architecture of core and variable chaperones. Nat Commun.

[50] Tobaben S, Thakur P, Fernández-Chacón R, Südhof TC, Rettig J, Stahl B (2001). A Trimeric Protein Complex Functions as a synaptic chaperone machine. Neuron.

[51] Weile J, Roth FP (2018). Multiplexed assays of variant effects contribute to a growing genotype-phenotype atlas. Hum Genet.

[52] Starita LM, Ahituv N, Dunham MJ, Kitzman JO, Roth FP, Seelig G (2017). Variant interpretation: functional assays to the rescue. Am J Hum Genet.

[53] Niu Y, Ferreira Azevedo CA, Li X, Kamali E, Haagen Nielsen O, Storgaard Sørensen C (2022). Multiparametric and accurate functional analysis of genetic sequence variants using CRISPR-Select. Nat Genet.

[54] Merk A, Bartesaghi A, Banerjee S, Falconieri V, Rao P, Davis MI (2016). Breaking Cryo-EM Resolution barriers to facilitate Drug Discovery. Cell.

[55] Fernandez-Leiro R, Scheres SHW (2016). Unravelling biological macromolecules with cryo-electron microscopy. Nature.

[56] Johansson LC, Stauch B, Ishchenko A, Cherezov V (2017). A Bright Future for serial femtosecond crystallography with XFELs. Trends Biochem Sci.

[57] Webb B, Lasker K, Schneidman-Duhovny D, Tjioe E, Phillips J, Kim SJ (2011). Modeling of proteins and their assemblies with the integrative modeling platform. Methods Mol Biol.

[58] Senior AW, Evans R, Jumper J, Kirkpatrick J, Sifre L, Green T (2020). Improved protein structure prediction using potentials from deep learning. Nature.

