# Supplementary material for: Detection of autism spectrum disorder-related pathogenic trio variants by a novel structure-based approach
Source: Mol Autism. 2024 Apr 3;15:12. doi: 10.1186/s13229-024-00590-9 (PMC10988830; doi:10.1186/s13229-024-00590-9)
Supplement: Supplementary file 1 — Additional file 1: Supplementary Table 1 Comparison of predictions from our model vs. Polyphen2, FATTHM and Alpha Missense. Supplementary Fig. 1 Quantification of co-immunoprecipitation of TRIO mutants with RAC1. Supplementary Fig. 2 Immunoblots of co-immunoprecipitation of TRIO binding mutants with RAC1. Supplementary Fig. 3 Immunoblots of co-immunoprecipitation of TRIO stability mutants with RAC1. Supplementary Fig. 4 Immunoblots of co-immunoprecipitation of TRIO benign mutants with RAC1 [file 13229_2024_590_MOESM1_ESM.pdf]

Supplementary Information

Supplementary Table 1. Comparison of predictions from our model vs. Polyphen2, FATTHM and Alpha Missense

#	Variant	Our Model	E-Phys Data	Polyphen2	FATTHM	Alpha Missense	Alpha Missense Score
1	G1453W	Damaging	Damaging	PROBABLY DAMAGING with a score of 0.999 (sensitivity: 0.14; specificity: 0.99)	Tolerated	pathogenic	0.9996
2	Y1318G	Damaging	Damaging	PROBABLY DAMAGING with a score of 0.999 (sensitivity: 0.14; specificity: 0.99	Tolerated	pathogenic	0.9949
3	C1387W	Damaging	Damaging	PROBABLY DAMAGING with a score of 0.999 (sensitivity: 0.14; specificity: 0.99)	Tolerated	pathogenic	0.9992
4	E1304G	Damaging	Damaging	PROBABLY DAMAGING with a score of 1.000 (sensitivity: 0.00; specificity: 1.00)	Damaging	pathogenic	0.9874
5	E1299W	Damaging	Dominant Negative	PROBABLY DAMAGING with a score of 1.000 (sensitivity: 0.00; specificity: 1.00)	Damaging	pathogenic	0.9999
6	A1464W	Damaging	Benign	PROBABLY DAMAGING with a score of 1.000 (sensitivity: 0.00; specificity: 1.00)	Tolerated	pathogenic	0.9988
7	T1430W	Damaging	Damaging	PROBABLY DAMAGING with a score of 1.000 (sensitivity: 0.00; specificity: 1.00)	Tolerated	pathogenic	0.9996
8	Y1383A	Damaging	Benign	PROBABLY DAMAGING with a score of 1.000 (sensitivity: 0.00; specificity: 1.00)	Tolerated	pathogenic	0.9966
9	T1394A	Benign	Benign	This mutation is predicted to be POSSIBLY DAMAGING with a score of 0.479 (sensitivity: 0.89; specificity: 0.90)	Tolerated	benign	0.197
10	S1403F	Benign	Benign	This mutation is predicted to be POSSIBLY DAMAGING with a score of 0.845 (sensitivity: 0.83; specificity: 0.93)	Tolerated	benign	0.1864
**Model vs. Experimental Data (n=10)**		**80.00%**	-	**70.00%**	**60%**	**80.00%**	**-**

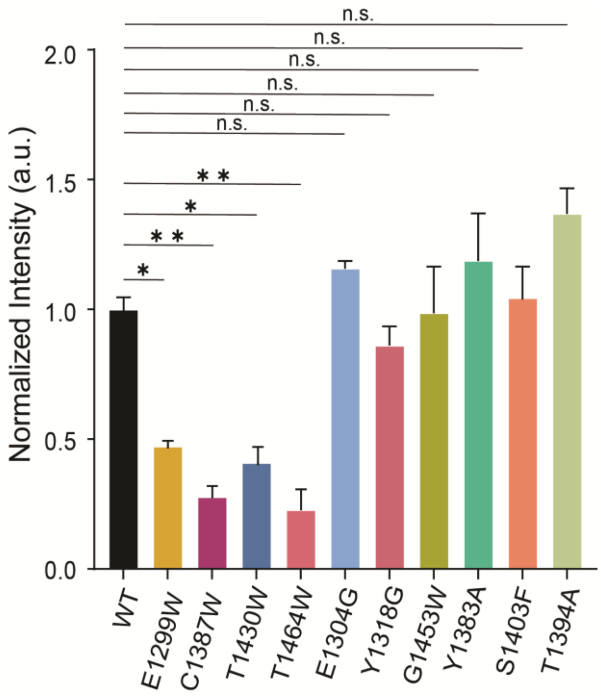

Supplementary Figure 1. Quantification of co-immunoprecipitation of TRIO mutants with RAC1. Barplot shows quantification of immunoprecipitated lysates from HEK293 cells co-expressing GFP TRIO constructs and FLAG-RAC1. Intensity of bands detected by anti-FLAG antibody were significantly lower in conditions with TRIO binding mutations (*p < 0.05, ** p < 0.01, n = 4 independent experiments, Shapiro-Wilcox test for normality, pairwise Student's t-test).

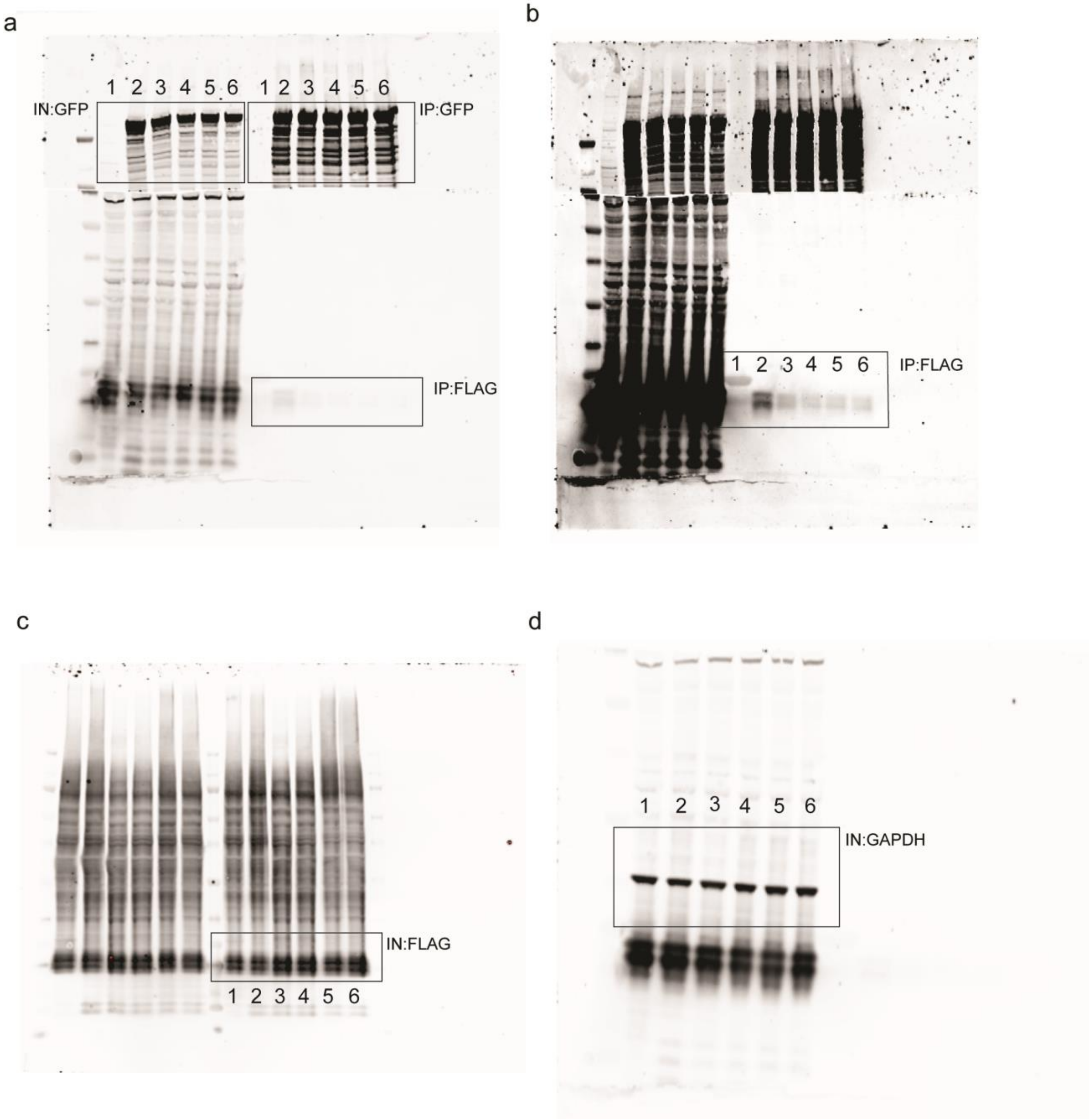

Supplementary Figure 2. Immunoblots of co-immunoprecipitation of TRIO binding mutants with RAC1. **a-d** Lanes indicated by 1-6 are respectively loaded equal amounts of lysates from HEK293T cells expressing GFP, GFP-Trio-WT, GFP-Trio binding mutants E1299W, C1387W, T1430W and T1464W respectively. **a**. Shows input and IP fractions probed with anti-GFP antibody (top), and anti-FLAG antibody (bottom, inset), shown at a higher exposure in **b.** Blots in **c.** and **d.** respectively show input fractions detected with anti-FLAG and anti-GAPDH antibodies.

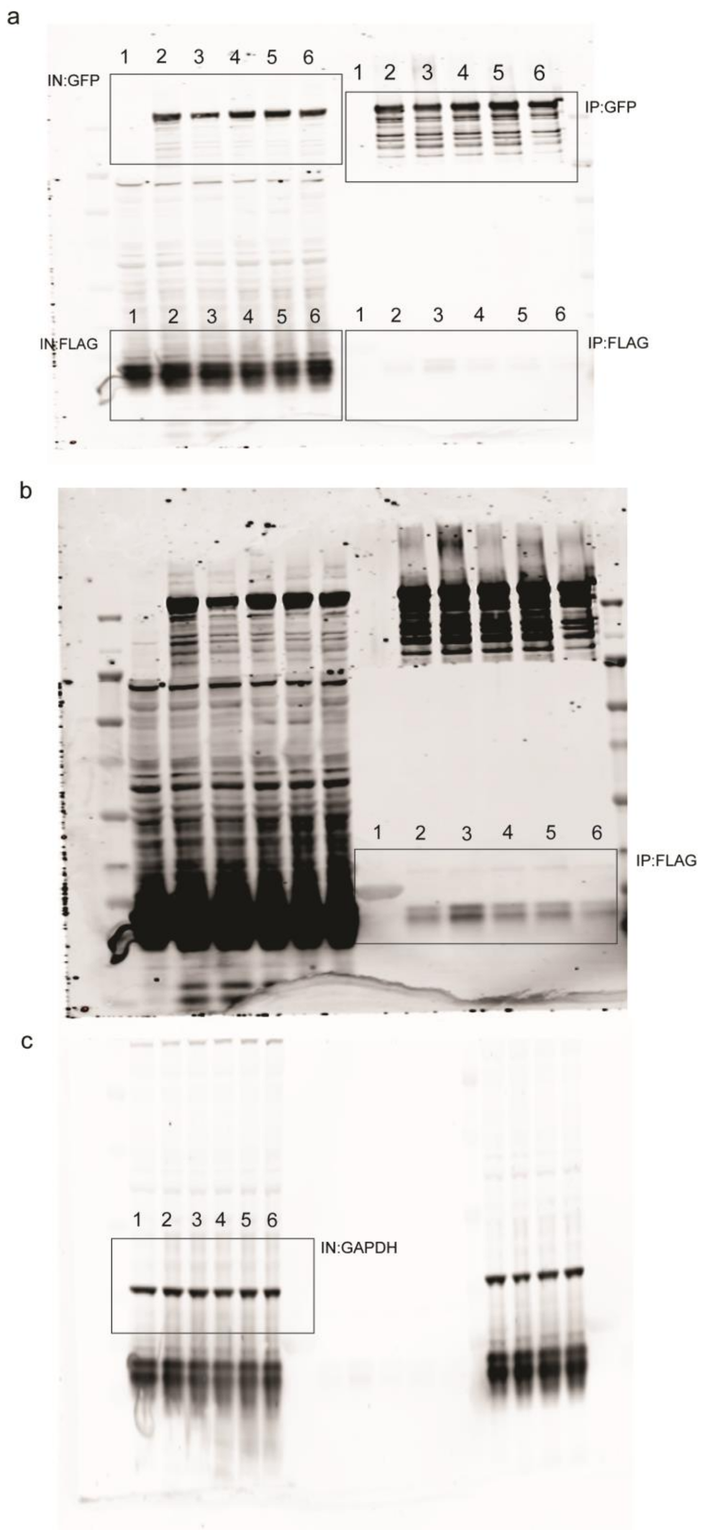

Supplementary Figure 3. Immunoblots of co-immunoprecipitation of TRIO stability mutants with RAC1. . **a-c** Lanes indicated by 1-6 are respectively loaded equal amounts of lysates from HEK293T cells expressing GFP, GFP-Trio-WT, GFP-Trio stability mutants E1304G, Y1318G, G1453W and Y1383A respectively. **a**. Shows input and IP fractions probed with anti-GFP antibody (top), and anti-FLAG antibody (bottom, inset), shown at a higher exposure in **b. c.** Box indicates input fractions detected with anti-GAPDH antibody.

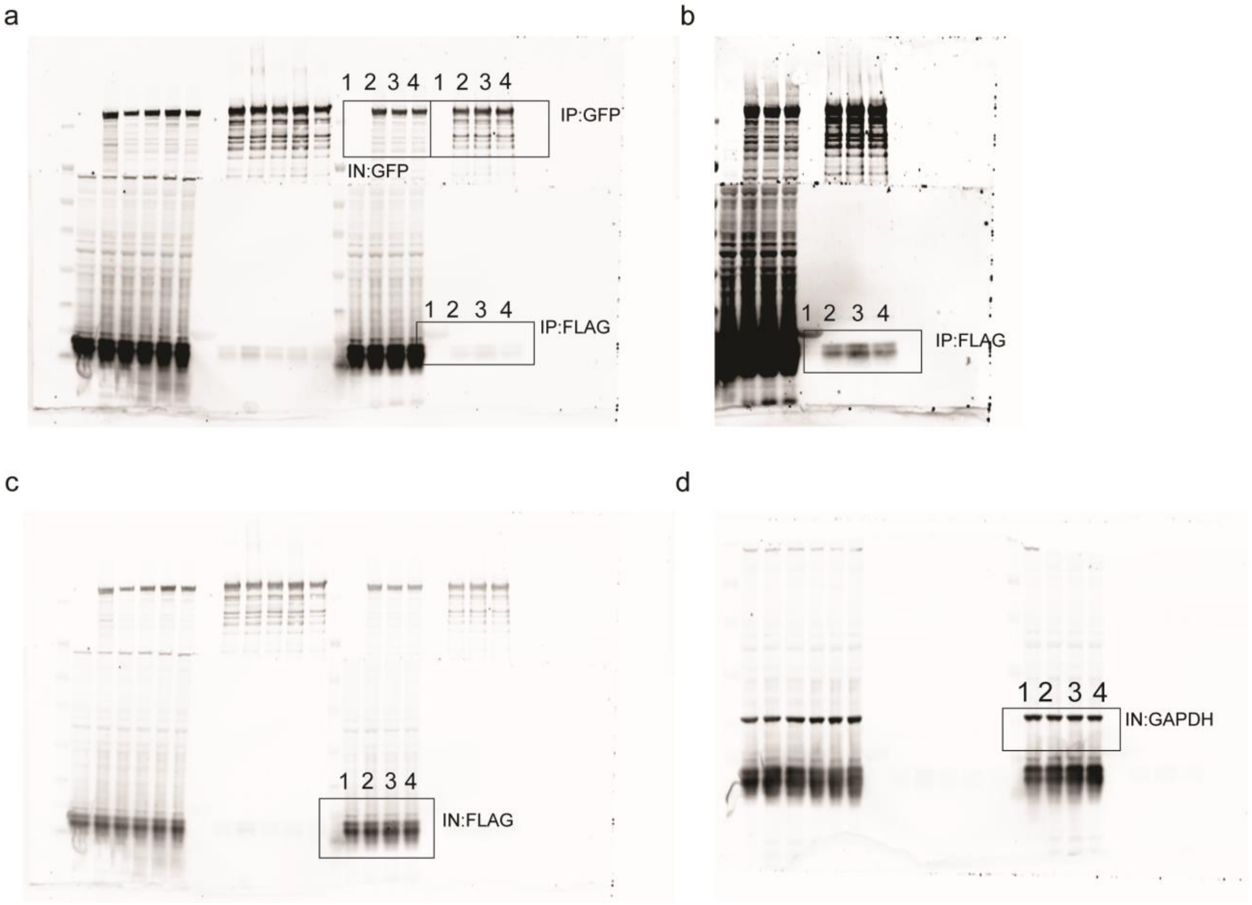

Supplementary Figure 4. Immunoblots of co-immunoprecipitation of TRIO benign mutants with RAC1.. **a-d** Lanes indicated by 1-4 are respectively loaded equal amounts of lysates from HEK293T cells expressing GFP, GFP-Trio-WT, GFP-Trio benign mutants S1403F and T1394A respectively. **a**. Boxes indicate input and IP fractions probed with anti-GFP antibody (top), and anti-FLAG antibody (bottom, inset), shown at a higher exposure in **b.** Blots in **c.** and **d.** respectively show input fractions detected with anti-FLAG and anti-GAPDH antibodies.
